# Heterocycles in Breast Cancer Treatment: The Use of Pyrazole Derivatives

**DOI:** 10.2174/0929867329666220829091830

**Published:** 2022-10-25

**Authors:** Sandra Ardevines, Eugenia Marqués-López, Raquel P. Herrera

**Affiliations:** 1 Laboratorio de Organocatálisis Asimétrica, Departamento de Química Orgánica. Instituto de Síntesis Química y Catálisis Homogénea (ISQCH), CSIC-Universidad de Zaragoza. C/Pedro Cerbuna 12, E-50009 Zaragoza, Spain

**Keywords:** Breast, cancer, heterocycle, pyrazole, tumor, HDI

## Abstract

Among the aromatic heterocycle rings, pyrazole –a five-membered ring with two adjacent nitrogen atoms in its structure has been postulated as a potent candidate in the pharmacological context. This moiety is an interesting therapeutic target covering a broad spectrum of biological activities due to its presence in many natural substances.

Hence, the potential of the pyrazole derivatives as antitumor agents has been explored in many investigations, showing promising results in some cases. In this sense, breast cancer, which is already the leading cause of cancer mortality in women in some countries, has been the topic selected for this review, which covers a range of different research from the earliest studies published in 2003 to the most recent ones in 2021.

## 1 INTRODUCTION

It is well known that cancer is the second cause of mortality in the world after cardiovascular diseases [1]. Every year, millions of people get this disease, and half lose their lives. Although chemotherapy is one of the most effective and extensive treatments to combat this malady, the lack of selectivity of the drugs joined to the secondary effects caused by them or the development of drug-resistance experimented by the patients reduces the efficacy of this approach [2]. Consequently, this supposes a big problem of health in many developed countries, and a great number of research groups have focused their efforts on the study of this mortal illness [3-12]. Therefore, there is a current need for discovering new techniques and drug candidates overall due to the high mortality rates [13-15].

### 1.1 Breast Cancer

Among the different types of cancer, which overall represent approximately 30% of premature deaths among the adult population aged 30-69 [16], breast cancer is postulated to be the predominant form of cancer in the female population, involving more than 50% of the reported cases of this disease [17].

Although hereditary and genetic factors such as personal or family history of breast or ovarian cancer and inherited mutations (in BRCA1, BRCA2 and other breast cancer susceptibility genes) account for 5-10% of breast cancer cases, studies in relation to migration processes have shown that non-hereditary factors are the main drivers of international and inter-ethnic differences. This has been demonstrated in comparative studies of low-risk populations that have migrated to high-risk populations, the movement of which has led to increased breast cancer incidence rates in subsequent generations [18].

These increased incidence rates in countries with higher HDI (Human Development Index, a summary measure of average achievement in dimensions of human development: a long and healthy life, being knowledgeable and having a decent standard of living) are associated with a higher prevalence of known risk factors. On the other hand, high incidence rates in transition countries are related to a higher prevalence of known risk factors concerning menstruation, reproduction, nutrition or anthropometry, among others [19].

However, knowledge of this disease in terms of geographical or temporal variations in rates and specific etiological factors is still limited. Looking at different geographical areas, it can be observed that countries, where incidence rates have historically been low have experienced a marked increase during the last decades (*e.g.*, in South American, African or Asian countries) [20]. These trends are probably due to a combination of demographic factors related to social and economic development. Similarly, among other aspects, the decline in incidence in the early 2000s in countries such as the United States, Canada, the United Kingdom and France was attributed to a decrease in the use of postmenopausal hormone treatment following the report of the Women's Health Initiative study linking the use of postmenopausal hormones with an increased risk of breast cancer [21].

The major risk factors for breast cancer are not easily modifiable because they derive from endogenous and prolonged hormonal exposures, although prevention by reducing alcohol consumption, engaging in physical activity, enhancing breastfeeding during the postpartum period or limiting hormone therapy may be beneficial.

### 1.2 Heterocycle-Based Drugs

**Fig. (1):**
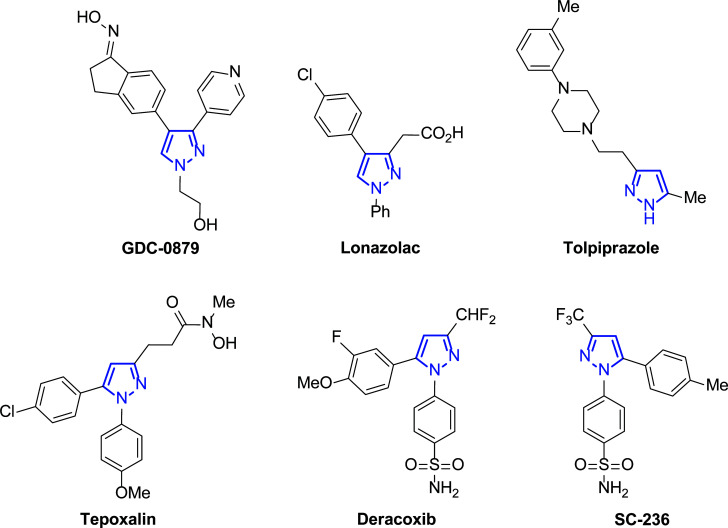
Examples of pyrazole-based biologically active compounds.

Within the plethora of anticancer drugs, those based on heterocycles have received special attention [22-27]. Among them, the pyrazole core is present in many of these potential active compounds (Fig. **1**), and new synthetic methods have been developed in the last two decades to achieve a great scope of this interesting family of targets [28].

In view of its large pharmacological scope, the pyrazole skeleton is considered a privileged scaffold in medicinal chemistry because of its properties as anti-influenza [29], antiviral [30-32], anti-inflammatory [33], antitubercular, antimicrobial, antioxidant, antidepressant, and insecticidal agent [34-36]. Related to cancer treatment, Crizotinib and Ruxolitinib [37] or Celecoxib [38-40] are important pyrazole-based anticancer drugs [41, 42]. Interestingly, there are also pyrazole-based drugs commercially available such as those described in Fig. (**2**) [43], among others.

Like many other drugs, pyrazoles exhibit activity due to their inhibitory capacity of diverse pivotal targets directly involved in the mechanism of cancer. In this work, we will also consider different aspects related to the role of pyrazole in the mechanism of cellular death. It is also remarkable that the rational design of new pyrazole scaffolds could be of interest in new drug discovery.

We would like to give the readers a broad overview of the plethora of pyrazole structures synthesized in the last two decades (2003-2021), emphasizing their utility as privileged scaffolds in the context of breast cancer, as previously mentioned, one of the most widespread cancers in women [44]. In this way, various representative examples of highly substituted pyrazole scaffolds and their pharmacological activity as potential anticancer drugs are illustrated and discussed in this work.

## 2 PYRAZOLES AS PROMISING ANTITUMOR AGENTS AGAINST BREAST CANCER

Hereafter, a great number of studies related to the different pyrazole derivatives with interesting results as antitumor agents are reported. Some of the sections in which this review has been structured are their capacity as kinase inhibitors, angiogenic agents, and those that act on estrogen-dependent factors or studies based on the synergistic effect with other structures of interest. Although the tumor lines studied in most research are diverse (and will be mentioned), only the results focused on breast cancer cells will be highlighted in this revision.

### 2.1 Role as Kinase Inhibitors

Biological functions in eukaryotes are regulated by phosphorylation and dephosphorylation of proteins by kinases and phosphatases, respectively [45]. Therefore, the blocking of kinases is considered for possible therapies against cancer as well as inflammatory diseases. Hence, the human genome encodes many of these proteins, some of which are recognized as disease-relevant, and small molecules targeting the ATP-binding site in these enzymes may inhibit the phosphorylation selectively, despite its ubiquitous nature [46, 47].

Moreover, selective inhibitors can be modelled by optimizing the peripheral groups, with the pyrazole moiety being a favored structure targeting kinases [48-51]. Over the last decade, much research has highlighted the use of pyrazole derivatives as kinase inhibitors with a broad spectrum of biological properties with a high pharmacological interest in different areas, like those referred to antimicrobial activity and neurodegenerative disorders or even cancer, among others [52].

In this context, Nielsen and co-workers carried out the synthesis of highly functionalized pyrazole carboxamides and carboxylic acids by saponification and transamidation, respectively, of ester-functionalized pyrazoles [53]. Following this synthetic protocol, pyrazole scaffolds were tuned to optimize protein kinase inhibition, being tested on AKT1, CK2, PKA, PKCα and SAPK2a (p38) (each compound was studied using a final concentration of 10 μM). The most promising inhibitor compounds are depicted in Fig. (**3**). Thus, ester **1a** showed significant inhibition of AKT1 and SAPK2a (p38) kinases, reducing activity by 40% and 35%, respectively (Fig. **3A**), while carboxylic acid **1b** inhibited AKT1 by 46% and PKA by 82% (Fig. **3A**). Additionally, the most promising demonstration of inhibitory activity was observed for amide **1c** with a lipophilic attachment, which inhibits several kinases (AKT1 by 61%, CK2 by 42% and PKA by 84%), indicating its multi-targeting approach (Fig. **3**, **A**).

It is known that a drug targeting, for instance, AKT, is expected to affect cell growth and viability. Therefore, to further evaluate the physiological effect of these compounds, the authors first analyzed human MCF-7 breast cancer cells by phase-contrast microscopy either untreated or incubated for 48 h with the tested product at concentrations of 50 and 100 μM. The morphological effects revealed that treatment with **1a** and **1c** had a clear inhibitory effect on cell growth (especially at the higher concentration), while treatment of the cells with **1b** caused no change in cell growth (Fig. **3B**, (**a**)). However, since no large numbers of floating dead cells were observed under the microscope for none of the compounds, it seems they did not cause severe cell death.

Later, the crystal violet binding method was used to quantify the effect of the selected inhibitors on cell growth (Fig. **3B**, (**b**)). Then, incubation of cells for 24 h with ester (**1a**) at 50 and 100 μM concentrations resulted in a ∼50% decrease in viability, reaching up to 24% of cells remaining with 100 μM after 72 h; although the best result corresponded to amide **1c**, which induced the loss of 90% of cells under the same conditions (100 μM after 72 h). However, carboxylic acid **1b** did not lead to a significant alteration. Since crystal violet staining does not distinguish between actively dividing and quiescent cells, the reduction in cell numbers could result from cytotoxic and cytostatic effects.

Moreover, the distribution of cells was analyzed in different phases of the MCF-7 cell cycle before and after treatment with 100 μM of these pyrazole derivatives for 24 h and 48 h (Fig. **3B**, (**c**)). In this case, only incubation with **1c** for 48 h produced cell cycle arrest (S phase: 7.7% (control) → 20.3% (treated cells) and G2/M phase: 11.1% (control cells) → 22.4% (treated cells)), while the other two compounds **1a** and **1b** did not cause any variation in it. According to these data, the effect of amide **1c** over cell viability can be attributed to arrested cells, while ester **1a** influence can be associated to deceleration of cell metabolism.

In a study reported in 2010 by Lee’s group, 1*H*- and 2*H*-pyrazole derivatives were designed and synthesized for an extensive structure-activity relationship study [54] on the bases of the potent B-Raf inhibitor GDC-0879 (Fig. **1**) [55]. In Fig. (**4**), six compounds out of thirteen (**2a-f**) showed very good activity in preliminary assays at 10 μM and were derived in subsequent assays to determine the IC_50_ in 60 cell lines. In particular, excellent values were obtained for six breast cancer cell lines (Fig. **4**), which in some cases were in the nanomolar range (*i.e.*, 26 nM for **2c** in MDA-MB-468).

The results obtained from the *in vitro* assays, together with the structure-activity relationship (SAR) correlation studies, revealed that to obtain the best activity, the compounds should be substituted at the 4-position of the pyrazole with a 4-(dimethylamino) phenyl group (**2a**, **2c** and **2d**) at the 2-position of the terminal pyridyl group, followed by 4-phenoxyphenyl (**2e**) and 4-acetyl (**2b**) substitution. The acetonitrile group at the 2-position of the pyrazole ring (2*H* isomers) was also enhanced over the 1-position. In addition, the effect of demethylation of the methoxy group (**2a**) to produce the hydroxyl analogues (**2d-e**) was not constant in the derivatives, suggesting the possibility of variable binding modes of these compounds at their target site or the presence of different targets, whose binding mode at each molecule would be different.

Moreover, following the interest in kinase inhibition and the understanding of the mechanism of action, compound **2c**, which had the highest potency in almost all cancer cell lines, was tested at 10 μM concentration on a panel of 54 kinases at Reaction Biology Corporation, showing very good selectivity for FLT3 kinase with an inhibition percentage of 82.3%. This would mean that this compound could be used as a good new lead for developing selective FLT3 kinase inhibitors [56-58]. However, the moderate potency of the compound on FLT3 kinase (IC_50_ = 1.74 μM) could not justify its strong and broad-spectrum anticancer activity, and this suggested the presence of other underlying mechanisms that (in addition to moderate FLT3 kinase) might control the anticancer activity.

As well-known, the epidermal growth factor receptor (EGFR) kinase plays a crucial role in signal transduction pathways that regulate cell division, differentiation and migration. As usual, its abnormal function implies the development of illness. Thus, the overexpression of EGFR has been observed in many human cancers, such as bladder, breast, colon and lung cancers [59, 60]. In this context, small molecules may inhibit the kinase activity of EGFR by binding the ATP site of the intracellular tyrosine kinase domain to block tyrosine kinase (TK) autophosphorylation. This is a traditional area of research for developing anticancer drugs [61, 62] and many EGFR inhibitors have been used to treat human cancers [63-65].

Among other interesting structures, compounds containing pyrazole and cinnamyl moieties have shown high inhibitory activity against EGFR [66-70]. In particular, cinnamic acid ester derivatives have been postulated as structures with potential antitumor activity [71, 72]. For instance, Qian and co-workers found some metronidazole derivatives **3** to be potent inhibitors of EGFR and HER-2 (Fig. **5**), as well as they presented good antiproliferative activity against MCF-7 cell line, such as compounds **3a**-**b** which reached values of IC_50_ of 0.36 and 0.98 μM, respectively [73].

A few years later, the same group performed the synthesis and biological evaluation of other series of pyrazole-cinnamoyl derivatives **4** (Fig. **6**) [74]. Antiproliferative activity against MCF-7 (breast cancer) of the synthesized compounds was also tested in this case, showing remarkable effects. In addition, it was found that most of these derivatives presented good inhibitory activity against EGFR and HER-2. Among them, compound **4ae** exhibited the most potent activity (IC_50_ = 0.30 μM (MCF-7), IC_50_ = 0.21 μM (EGFR) and IC_50_ = 1.08 μM (HER-2)) (Fig. **6**).

Structure-activity relationship studies were performed by modification of the parent compound to determine how these substituents might affect the activity. Inspection of the chemical structures of compounds suggested that they could be divided into two subunits: The A-ring and the B-ring (Fig. **6**). The introduction of an electron-donating group in both could enhance its antiproliferative activity, and the order of potency was OMe > Me. At the same time, compounds with electron-withdrawing groups in ring B caused a decrease in the activity.

The authors further performed a molecular modeling study to explore the interaction with the active site of EGFR/HER-2, taking as a reference compound **4ae**, which had provided the best inhibition values. It was found to bind very efficiently to the ATP binding site of EGFR through hydrophobic interactions and stabilized by a hydrogen bond and a π-σ interaction. Thus, the oxygen atom (OMe group) at the A-ring was able to form a hydrogen bond with the amino hydrogen of Lys A: 721 (N-HꞏꞏꞏO = 2.477 Ȧ; 130.6˚). A π-σ interaction between Leu694 and the benzene ring (bond length: 2.48 Ȧ) was also confirmed. Similarly, it was seen that compound **4ae** bound to the active site of HER-2 *via* hydrogen bonding through the formation of a hydrogen bond between that oxygen atom and the amino hydrogen of Met A: 801 (N-HꞏꞏꞏO = 2.278 Ȧ; 145.8˚).

Among the most recent research, Shahsavari’s group has focused on triple-negative breast cancer [75], which is approximately 15-20% of all breast carcinomas and is associated with an earlier age of onset, an aggressive clinical course and a poor prognosis [76].

During this study, the activity of a series of 1,3-diaryl-5-(3,4,5-trimethoxyphenyl)-1*H*-pyrazoles (**5**) and 1,3-diaryl-5-(3,4,5-trimethoxyphenyl)-4,5-dihydro-1*H*-pyrazoles (**6**) was evaluated against MDA-MB-468, the triple-negative human breast cancer cell line, and the *in vitro* cytotoxicity assays revealed its dose- and time-dependent anti-proliferative properties (Fig. **7**). 3-(4-Methoxyphenyl)-1-(*p*-tolyl)-5-(3,4,5-trimethoxyphe-nyl)-4,5-dihydro-1*H*-pyrazole (**6f**) resulted in being the most active compound against MDA-MB-468, with IC_50_ values of 14.97 μM and 6.45 μM, after 24 and 48 hours, respectively, compared to the positive control (Paclitaxel, IC_50_ values of 49.90 μM and 25.19 μM). This compound also induces the minimum toxicity on normal fibroblast cell lines (AGO1522), with IC_50_ values of 28.74 μM and 20.33 μM, after 24 and 48 hours, respectively.

Additionally, a cell cycle study was performed after treatment with 14.97 μM of the most active compound (**6f**) after 24 h, concluding that the arrest occurred during the S phase. The cell death assays determined a significant increase in the early apoptotic phase in treated cells, concluding that apoptosis, and not necrosis, was the mechanism triggered by these compounds. Once this was known, the role of reactive oxygen species (ROS) in the induction of apoptosis was studied. The results of these assays led to the conclusion that ROS plays an important role in the apoptosis process. In addition, the fluorometric assay kit was used to measure the activity of caspase 3 as the main protease in the signaling of the apoptosis process, and a significant increase in its activity was observed in the cells treated with the candidate compound **6f** compared to the control.

### 2.2 Estrogen Dependant Anti-Cancer Derivatives

**Fig. (2):**
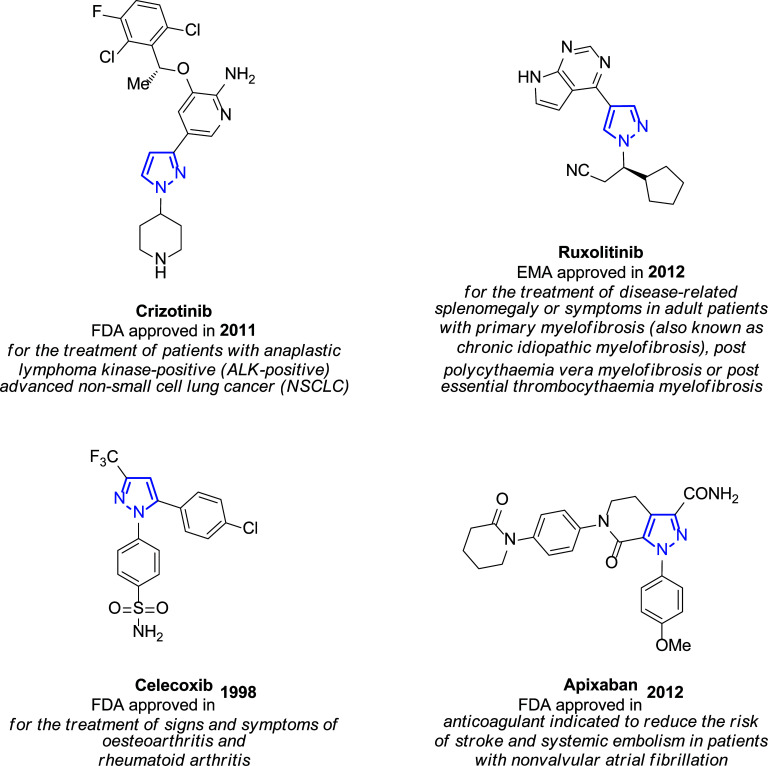
Examples of pyrazole-based drugs are approved for commercialization [43].

**Fig. (3):**
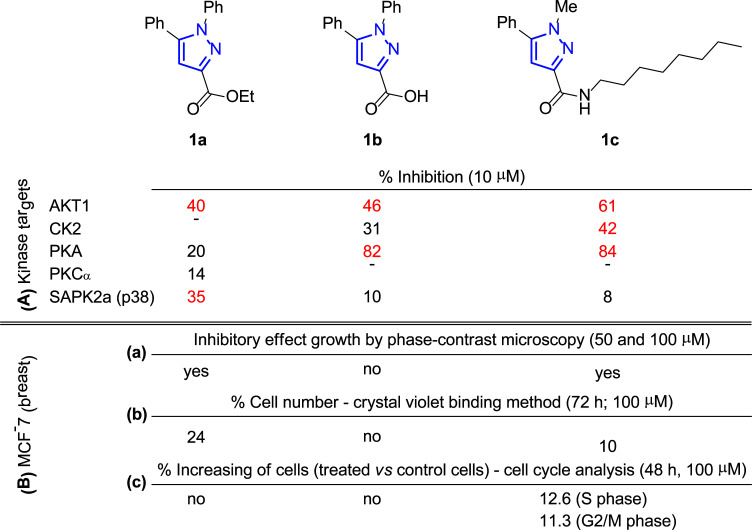
Pyrazoles **1** with protein kinase inhibition (**A**) and antitumoral activity (**B**) [53].

**Fig. (4):**
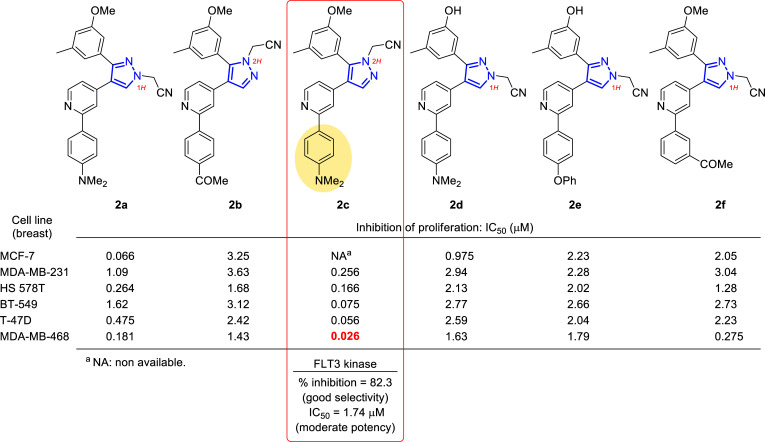
Selected compounds **2a-f** to carry out the 60-cell-line screening at the National Cancer Institute (NCI), Bethesda, Maryland, USA and IC_50_ values in μM for six different cell lines of breast cancer [54].

**Fig. (5):**
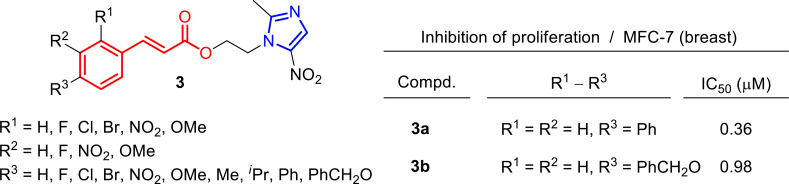
Cinnamic acid metronidazole ester derivatives **3** were evaluated as anticancer agents, and IC_50_ values for the two most active molecules (**3a**-**b**) against the MCF-7 cell line [73].

**Fig. (6):**
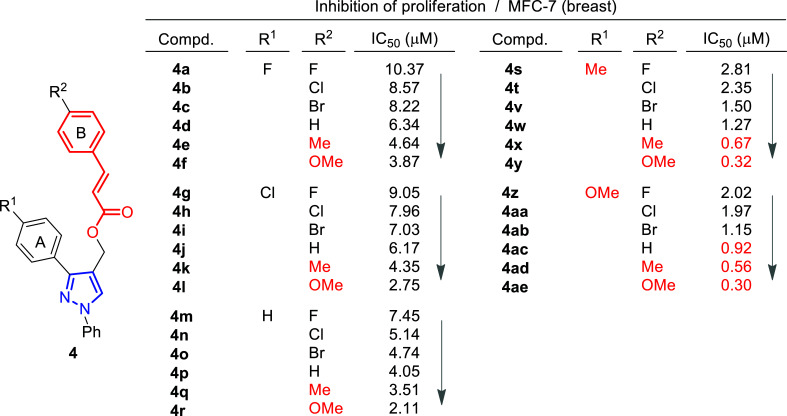
Structure-activity relationship study of pyrazole-cinnamyl derivatives **4** [74].

**Fig. (7):**
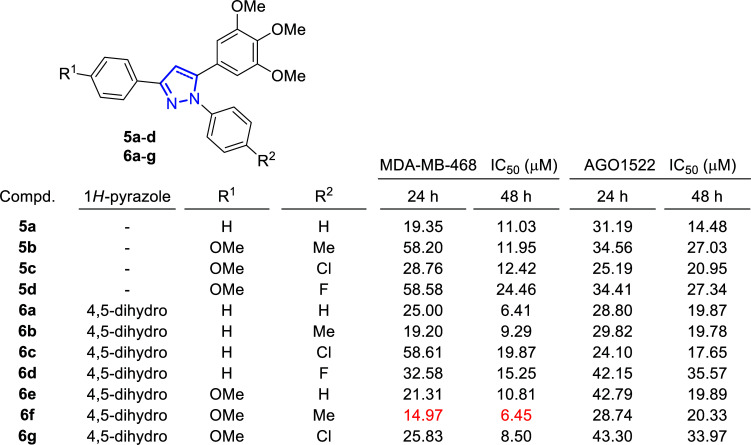
Inhibitory growth on breast cancer cells (MDA-MB-468) and toxicity on normal fibroblast cell line (AGO1522) of synthesized compounds **5a**-**d** and **6a**-**g** [75].

Estrogens control the normal function of female sex organs, secondary sex characteristics, and mammary glands. Moreover, some breast cancers are estrogen-dependent, where the binding of estrogens to their receptors activates the transcription of their target genes, the last ones responsible for the proliferation of cancer cells in the tumor [77]. In this context, therapy based on anti-estrogens is used in several diseases, such as breast malignancies [78], including estrogen receptor antagonists, such as tamoxifen [79, 80] and toremifene [81, 82]. The discovery of the effect of cyclooxygenase-2 (COX-2) inhibitors, such as celecoxib (Fig. **2**) or meloxicam [83] in many types of malignancies, especially in breast cancer [84-86], made 1,5-diphenylpyrazoles became important structures to work with.

**Fig. (8):**
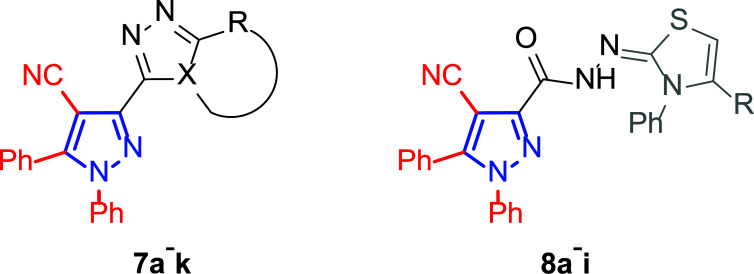
General structures of 4-cyano-1,5-diphenylpyrazole derivatives **7** and **8** evaluated [87].

Motivated by this background, Farag and co-workers were interested in synthesizing several 4-cyano-1,5-diphenylpyrazoles linked to different heterocyclic ring systems (**7a**-**k**) and acyl hydrazide derivatives (**8a**-**i**) at the 3-position (Fig. **8**) to test their anti-estrogenic effects *in vivo* and to evaluate their cytotoxic properties against estrogen-dependent tumors *in vitro* [87].

The *in vitro* assays were performed on seven breast tumor cell lines, namely MDA/MB-231 ATTC, MCF-7, T-47D, NCI/ADR-RES, HS-578T, MDA/MB-435, MiDA-N and BT549, as well as on additional ovarian cancer cells (6 lines), which were also addressed in this study.

Tables **1** and **2** collect the results as growth inhibitory concentration (GI_50_) values for all the breast cancer lines tested, showing good activity (below 10 μM) in red. Regarding the effect of the structure, some correlations were found and are summarized as follows. Analyzing first the results obtained for a big family of triazoles (**7a-i**, Table **1**), it is worth mentioning how variations on mercaptotriazole derivative **7a** affect, which revealed good activity (2.48-6.68 μM) against five out of eight cell lines. Concerning the substitution on the N, increasing lipophilicity with a phenyl group (**7b**) resulted in total inactivity, while a more hydrophilic substituent, such as an amino group (**7c**), improved the activity against only one of the cell lines (HS-578T, 8.94 μM) for which compound **7a** was inactive.

Additional changes on the inactive molecule **7b**, such as methylation of the thiol group (**7d**), caused little improvement in the activity, getting GI_50_ up to 36.3 μM, whereas produced no effect in the case of replacement of the thiol group by a hydrazine moiety (**7e**), which was also totally inactive. The idea of generating fused ring systems (**7f**-**i**) out of the amino and thiol groups of molecule **7c** had different effects depending on the cell line. Hence, in the case of the two derivatives with a second 5-membered ring (**7h**-**i**), aromatic substitution (Ph) rendered a totally inactive compound (**7h**), while aliphatic substitution (Bn) involved improvement only for two of the cell lines (MDA MB-231 ATTC and NCI ADR-RES), keeping similar activity for the other ones. The other two derivatives with a second 6-membered ring (**7f**-**g**) both contain aromatic substitution; a curious complementarity is observed in the case of a phenyl group (**7f**), compared to parent molecule **7c**, meaning that the most sensitive cells (HS-578T, MDA MB-435 and MiDA-N) for the later (**7c**), are the most resistant ones for the former (**7f**). Exactly the same occurs when the comparison is between **7f** and **7i**. In general, introducing a methoxy group in the *para*-position of the phenyl substituent (**7g**) translates into the best activity, except against MCF-7, T-47D and BT549, compared to both its parent molecule **7c** and its homologous **7f**. In fact, the best activity within all newly synthesized compounds is achieved in derivative **7g** with GI_50_ values of 1.19 and 1.23 μM against MiDA-N and MDA MB-435, respectively.

Finally, the change of the N of the triazole by another heteroatom was examined. In this context, the activity of oxadiazole **7k** is worst compared to its homologous triazole **7a**, except against MCF-7, HS-578T and BT549. Thiadiazole **7j**, which is not exactly comparable with **7a** because of its different substitution at the C-position (**7j**: NHPh and **7a**: SH), gave good inhibition in the case of MDA MB-231 ATTC and MCF-7 (5.84 and 8.53 μM, respectively).

The second line of structural optimization was carried out based on the remarkable cytotoxicity of the carboxylic acid hydrazide derivative **8a** (GI_50_ 1.34 μM against MiDA-N, Table **2**). Thus, for instance, the thiosemicarbazide derivative **8b** was found to have moderate to weak activity, whereas the potassium salt **8c** provided good inhibition between 2.86 and 9.30 μM against MDA MB-231 ATTC, HS-578T, MDA MB-435 and MiDA-N cell lines.

Additionally, the pyrazole derivative **8d** seemed cytotoxic against MDA MB-231 ATTC, T-47D, NCI ADR-RES and MDA MB-435 lines (6.21, 2.45, 2.76 and 2.75 µM, respectively), while in general, its homologous pyrazolone **8e** had a moderate to weak cytotoxicity.

Finally, the authors also explored incorporating a 5-membered ring containing S and N in the 2-N position of the acyl hydrazide (**8f**-**i**). Thus, the integration of phenylthiazolidene led to compound **8f** with the broadest scope, showing GI_50_ values ranging between 4.12 and 8.62 µM for all the cell lines except T-47D (62.5 µM) and either replacing the phenyl group with a methyl group (**8i**) or incorporating a chlorine atom at the *para*-position of it (**8g**) allowed the improvement of the activity against this cell line (26.5 and 22.2 µM, respectively), being the inhibition worse for the rest of tested cell lines. Curiously, substitution in the *para*-position of the phenyl ring with a methoxy group turned the compound (**8h**) inactive.

Acute *in vivo* toxicity studies were performed on a representative sample of the novel compounds synthesized. Rats were orally treated with different doses of an aqueous suspension of a very fine powder of the tested compounds. Under these conditions, as shown in Table **3**, the tested compounds were found to be highly safe (LD_50_ values) for albino rats compared to the reference drug letrozole [88].

Additionally, many pathological complications (breast cancer, endometrial cancer, osteoporosis, *etc.*) originate in aberrant expression of estrogen receptor alpha (ER-α). It plays a key role in breast cancer initiation and progression and confers chemoresistance to cancer cells by regulating the expression of anti-apoptotic proteins (Bcl2) [89]. The overexpression of estrogen receptors is responsible for 70% of breast cancer cases, named estrogen receptor-positive tumors [90, 91].

**Fig. (9):**
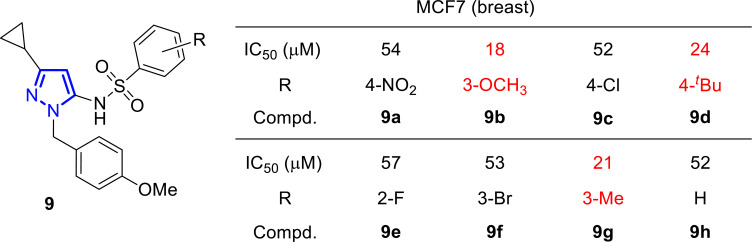
1-(4-Methoxybenzyl)-3-cyclopropyl-1*H*-pyrazol-5-amine derivatives **9a**-**h** and IC_50_ (μM) values against MCF-7 [92].

On the base that pyrazoles are good ligands for estrogen receptors, Rangappa and coworkers have been working in the last years on the synthesis and study of the antitumoral activity of novel derivatives. From a series of novel 1-(4-methoxybenzyl)-3-cyclopropyl-1*H*-pyrazol-5-amine derivatives **9a**-**h**, some compounds exhibited interesting growth inhibitory effects against MCF-7 cell line (Fig. **9**) [92]. Structure-activity relationship studies revealed that electron donating groups on the phenyl ring, such as 3-methoxy (**9b**), 3-methyl (**9g**), and 4-*tert*-butyl (**9d**), provided significant activity, whereas electrons withdrawing groups, such as 4-nitro (**9a**), 4-chloro (**9c**), 2-fluoro (**9e**) and 3-bromo (**9f**) delivered poor activity.

**Fig. (10):**
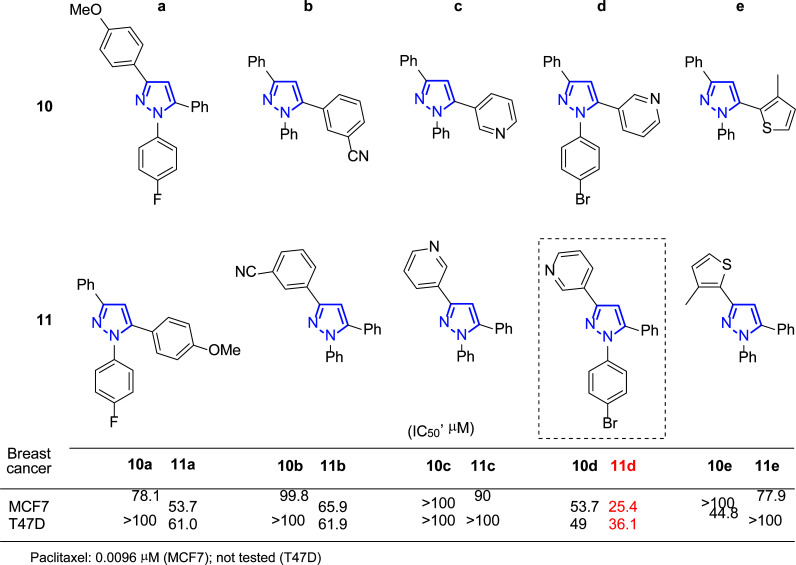
1-Aryl-3,5-bis(het)aryl pyrazole derivatives **10a**-**e** and **11a**-**e** and IC_50_ (μM) values against MCF-7 and T47D [93, 94].

Later in 2017, these authors prepared 1-aryl-3,5-bis(het)aryl pyrazole derivatives with complementary regioselectivity: **10a**-**e** *versus* **11a**-**e**, and also evaluated *in vitro* their activity against several breast cancer cell lines, such as MCF-7 and T47D (Fig. **10**) [93].

Compound **11d** showed the highest antiproliferative effects from this series, blocking cells in the sub-G1 phase of the cell cycle and promoting apoptosis. In addition, it was concluded that the compound decreased mitochondrial membrane potential, induced DNA fragmentation and chromatin condensation, and necessitated caspases cleavage in breast cancer cell lines [85].

Authors continued their work searching for new anti-estrogens or selective estrogen receptor modulators (SERMs) and published in 2018 further studies using compound **11d** (Fig. **10**) [94]. Molecular docking studies showed that fixes efficiently to the ligand binding domain of ER-α *via* hydrophobic interactions with a receptor pocket [95]. Additionally, *in vivo* assays were performed in Sprague-Dawley (SD) rats, carriers of a DMBA-induced mammary gland tumor. Twenty-five days after inoculation, animals were sacrificed, and the mammary glands were removed for evaluation. Treatment with the drug candidate reduced tumor size, indicating its ER-α antagonistic effect.

### 2.3 Pyrazoles with Anti-Angiogenic Activity

The growth of blood vessels (a process known as angiogenesis) is essential for many physiological procedures (embryogenesis, organ development, wound healing, *etc.*) [96, 97]. However, unregulated angiogenesis has been associated with different diseases, such as cancer, retinopathy, rheumatoid arthritis, psoriasis, atherosclerosis and hemangioma [98-101]. Antiangiogenic therapy targets activated endothelial cells and have gained increasing interest for its various advantages over targeted therapy. In addition, targets for anti-angiogenic therapy are easily accessible by systemic administration, as 10 out of 100 new tumor cells require at least one new endothelial cell (one gram of tumor contains approximately 20 million endothelial cells and 100 million to 1 billion tumor cells). Therefore, inhibiting angiogenesis and stopping the growth of an endothelial cell could have an amplified effect on tumor cells [102].

In this sense, and taking into account the good results achieved in terms of apoptosis (the programmed cell death that maintains the balance between cell survival and cell death) for some tumor cell lines previously reported using structures such as celecoxib and SC-236 (Figs. **1** and **2**) [103-105], Abadi and co-workers carried out in 2003 the synthesis of a battery of 1,3,4-trisubstituted pyrazole derivatives. With a similar substitution pattern, those new structures proved to be potential antitumors and antiangiogenic agents [106].

**Fig. (11):**
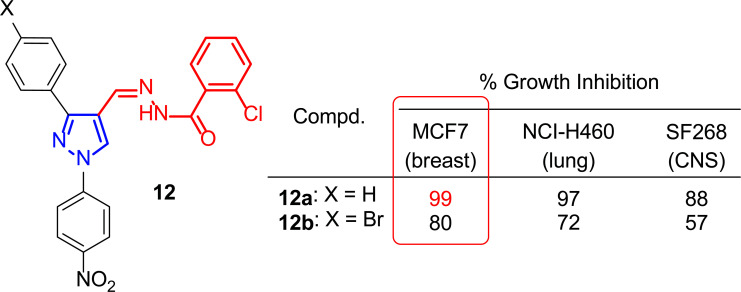
Selected compounds **12a**-**b** as the most active against MCF-7 cancer cell line [106].

The prepared compounds were evaluated in preliminary assays in 3 tumor cell lines (MCF-7 (breast), NCI-H460 (lung) and SF268 (central nervous system)) with a 10^–4^ M drug concentration, and the results were provided as a percentage of cell growth after 48 h of incubation. Of thirty-four compounds tested, **12a**-**b** (Fig. **11**) were the most active in MCF-7, being compound **12a** the best one which provided a cell growth inhibition of 99%.

A second test was performed for both compounds in *in vitro* screening, which provides the effect on cell growth in a panel of approximately 60 human tumor lines. These lines were divided into subpanels according to different representative tumor cell lines such as leukemia, ovary, prostate, breast or central nervous system. Although no comparatives were performed with normal cells, the ratio obtained by dividing the mean graph mid-point (MG-MID) (μM) of the full panel of the compound by the MG-MID (μM) of its subpanel is considered an indicator of selectivity. Ratios between 3 and 6 indicate moderate selectivity, while those above 6 indicate high selectivity towards the corresponding subpanel [107-109]. Compound **12a** exhibited significant anticancer activity with a broad spectrum of action, although without being selective towards a specific tumor line.

Furthermore, subsequent studies determined that these 1,3,4-trisubstituted pyrazole derivatives showed interesting antiangiogenetic activity, even in compounds that did not present cytotoxicity in the preliminary assays.

**Fig. (12):**
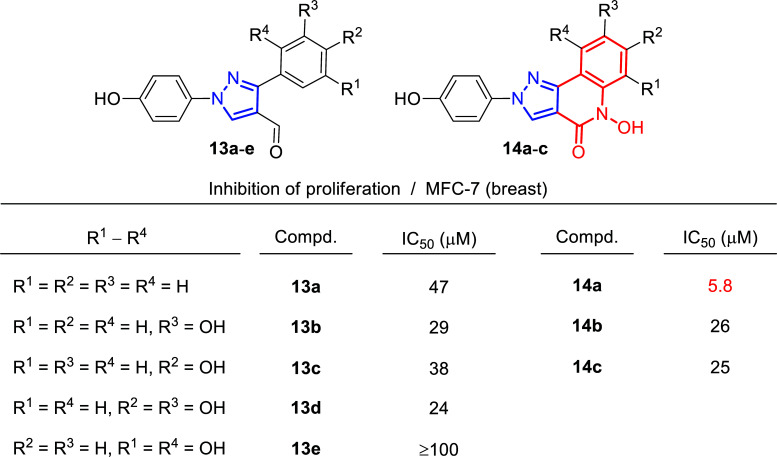
Cytostatic activity of compounds **13** and **14** [110].

In 2010 the group of Liekens and Haroutounian reported the synthesis of novel fused pyrazolo [4,3-*c*]quinolone derivatives as potential anti-angiogenic agents, combining in a single backbone the known pharmacophore quinoline ring with a substituted pyrazole. In the course of this investigation, the authors also evaluated the cytotoxicity against various tumor cell lines, including MFC-7 (breast) (Fig. **12**) [110]. Therefore, *in vitro* tumor cell proliferation inhibition studies were performed for hydroxyl pyrazolocarboxaldehydes **13a**-**e** and hydroxyl pyrazoloquinolin-4-ones **14a**-**c**. Notably, most of the tested compounds showed moderate IC_50_ values for the MCF-7 line between 20 and 50 μM, being compound **14a** the most promising, with an IC_50_ value of 5.8 μM.

**Fig. (13):**
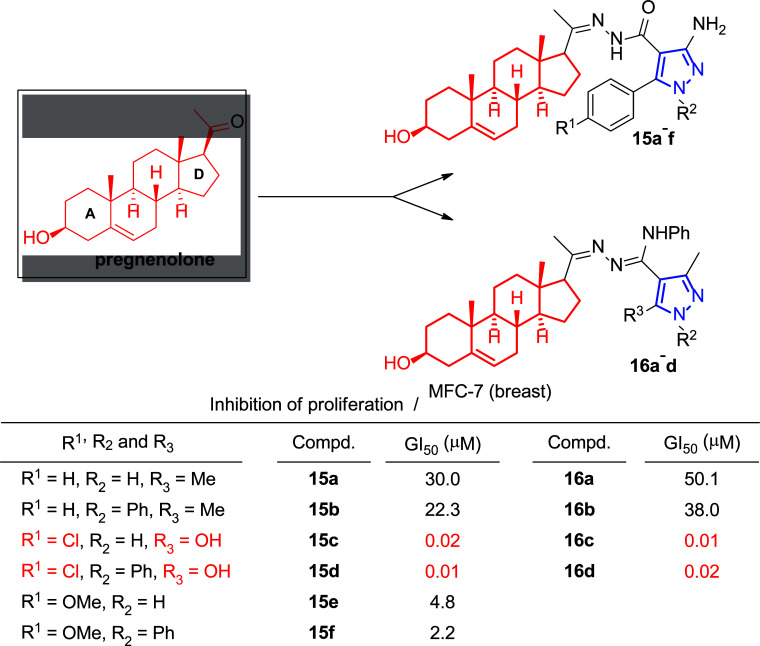
Pyrazole derivatives **15a**-**f** and **16a**-**d** synthesized from pregnenolone and their *in vitro* cytotoxic results [120, 121].

The advantages associated with steroid-based chemotherapeutics [111-113], including non-toxicity, reduced vulnerability to multidrug resistance (MDR) and high bioavailability, led to a growing interest in the modification of steroid molecules for their biological properties. This was demonstrated by different ring modification studies of steroid molecules involving the A- and D-rings, in which the incorporation of heteroatoms (N or O) was reported to enhance the biological activities of these molecules, including antimicrobial, anti-inflammatory, hypotensive, hypocholesterolemic and diuretic activities [114-117]. Among them, the natural steroid pregnenolone is a precursor of other hormones such as cortisone, estrogen, testosterone and progesterone [118, 119]. Under these conditions, in 2012, Mohareb’s group used it as a template to develop new anticancer compounds [120, 121]. Modification of the D-ring by reaction with cyanoacetylhydrazine gave rise to the hydrazone derivative (non-shown), which finally underwent heterocyclization reactions to give the pyrazoles **15a**-**f** (Fig. **13**), pyridine (non-shown), thiazole (non-shown) and thiophene (non-shown) derivatives of pregnenolone [120]. In a parallel article, the same authors modified this precursor by reaction with 1-phenyl-3-thiosemicarbazide to give the thiosemicarbazone derivatives (non-shown), which, under the same hypothesis as above, were subjected to heterocyclization reactions to give the derivatives thiazolyl hydrazonoandrostane (non-shown) and pyrazolyl semicarbazidoandrostane derivatives **16a**-**d** (Fig. **13**) [121].

The cytotoxicity of the newly synthesized heterocyclic steroids was studied against three human tumor cell lines, namely non-small cell lung cancer (NCI-H460), central nervous system cancer (SF-268) and breast adenocarcinoma (MCF-7). The *in vitro* cytotoxicity assays showed little selectivity towards the different tumor lines studied, although very good GI_50_ values were obtained. The data for the pyrazole derivatives of both studies against the MCF-7 line range between 0.01 and 50.1 μM, as shown in Fig. (**13**). In fact, some of the tested compounds reached the nanomolar range and, in some cases, showed much higher inhibitory effects towards the three tumor cell lines than the reference drug, doxorubicin (MCF-7: 0.04 μM). Regarding the structure-activity relationship, it is possible to make some comments. For instance, in the case of pyrazoles **15a**-**f**, it seems to be important the substitution of the phenyl group at the 5-position of pyrazole moiety, being the highly electronegative chlorine atom, the group that provided better GI_50_ values (**15c**-**d**: 0.02 and 0.01 μM), in comparison with the methoxy group (**15e**-**f**: 4.8 and 2.2 μM) and the unsubstituted phenyl ring (**15a**-**b**: 30.0 and 22.3 μM). On the other hand, in the case of pyrazoles **16c**-**d**, their higher inhibitory effect may be due to the presence of an electron-withdrawing OH group –again at the 5-position of pyrazole moiety– (0.01 and 0.02 μM), instead of a methyl group (**16a**-**b**: 50.1 and 38.0 μM).

### 2.4 Candidates with Anti-Invasive Effects

Up to now, cancer treatment has mainly focused on growth reduction. Surgery, radiotherapy, and chemotherapy have all proven to be very effective in tackling tumor volume. However, cancer patients usually do not die from growth effects but the sequelae of invasion and metastasis.

The molecular mechanisms of invasion are not completely understood, but important contributions have been ascribed to proteases secreted by cancer cells, which can degrade the surrounding extracellular matrix (ECM) to cancer cell motility factors and factors that disrupt cell-cell adhesion [122, 123]. In view of the molecular cross-talks between cancer cells and host cells, receptor signaling through cytoplasmic tyrosine and serine/threonine kinases has been recognized as an important study target in cancer research. It was this growing interest in tissue invasion, a hallmark of malignant tumors responsible for poor prognosis in untreated cancer patients, that encouraged the group of Parmar to search for a new anti-invasive treatment, screening a total of ninety-five compounds from different classes of heterocyclic scaffolds, including lactones, coumarins, chromans and pyrazoles, among others [124]. These derivatives were tested against organotypic confrontation cultures of invasive human mammary carcinoma MCF-7/6 cells with embryonic chicken heart fragments *in vitro*. The assay consisted of three-dimensional confrontations between tumor cells and normal tissue.

**Fig. (14):**
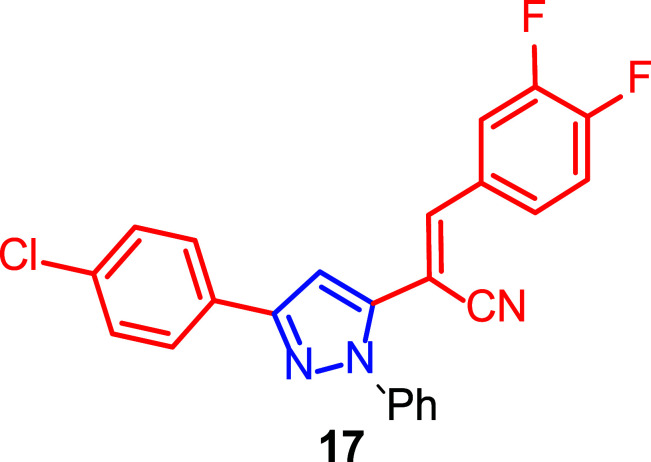
Pyrazole derivative **17** with anti-invasive activity [124].

In control cultures treated with solvent, MCF-7/6 cells invaded the PHF (precultured heart fragments) after 8 days of incubation. Many compounds showed no inhibition of invasion, and histologically their cultures were similar to controls. Other compounds were anti-invasive at the 10 µM concentration. However, only three of the 95 compounds, including pyrazol (**17**) (Fig. **14**), inhibited invasion of MCF-7/6 cells at a concentration as low as 1 µM. Unfortunately, none of the compounds was active at 0.1 µM. Furthermore, sulforhodamine B (SRB) assay showed no inhibition of MCF-7/6 cell growth by these three compounds, concluding that the compounds exhibited anti-invasive activity but were not cytotoxic to cancer cells.

Also, before performing the invasion assay on the synthesized compounds, several anti-invasive agents that inhibited both invasion and growth of tumor cells were screened. One of these compounds interfered with a common molecular target in mitosis and directional migration, namely vinca alkaloids, which bind to tubulin and prevent the assembly of the mitotic spindle and the cytoplasmic microtubule complex [125]. Another of the selected compounds was cytotoxic, with the anti-invasive effect resulting from a reduction in the number of intrinsically invasive cells in the challenged cultures.

However, the anti-invasive compounds in the study only limited invasion without affecting growth, which meant that their activity was not a result of cytotoxicity. These selective invasion inhibitors are structures of interest to understand which targets are involved in invasion mechanisms and which are involved in growth. The E-cadherin/catenin complex, for example, which is located in the cell membrane of normal epithelial cells, prevents epithelia from growing beyond their normal tissue boundaries [126]. In invasive tumors derived from epithelial tissues, the expression of this complex is down-regulated [127]. The complex in MCF-7/6 cells is inactive but is sensitive to functional regulation by several compounds shown to possess anti-invasive activity. Tangeretin, a citrus methoxyflavone, can increase E-cadherin-dependent cell-cell adhesion between MCF-7/6 cells and inhibit their invasion in the chicken heart invasion assay. This mechanism of action could be excluded for the anti-invasive compounds in the study, as no stimulation of cell aggregation was observed *in vitro*.

In summary, it was proved that the tested compounds did not inhibit tumor cell growth and did not have an effect through activation of the MCF-7/6 E-cadherin/catenin complex.

In addition, the broad battery of pyrazole-derived compounds synthesized allowed a further structure-activity study. Thirteen of the sixteen pyrazolyl acrylonitriles tested showed anti-invasive activity at 10 µM on MCF-7/6 cells in the chicken heart invasion assay. Moreover, among the five pyrazolyl acrylonitriles synthesized with the 3,4-difluorophenyl substituent at the 3-position, three of them showed anti-invasive activity at a concentration of 10 µM, but only compound **17** showed the activity at a concentration of 1 µM.

These results revealed that the difluorophenyl moiety at the 3-position and the 4-chlorophenyl moiety at the 5-position of the pyrazole in compound **17** enhanced the anti-invasive activity of acrylonitriles.

### 2.5 Synergistic Effects Related to Structure-Activity Relationship (SAR) Studies

**Fig. (15):**
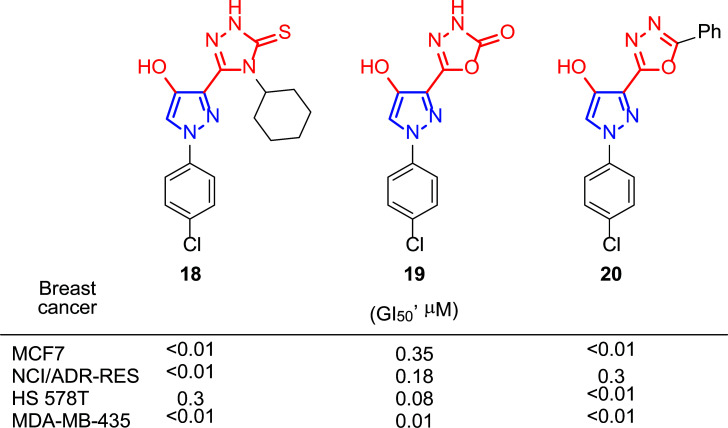
Compounds **18**, **19** and **20** proved to be the most active ones in Rostom’s study [128].

The growing interest in the biological activity of pyrazole derivatives and the study of pharmacophore groups in the cancer field led Rostom and co-workers to prepare compounds with a 4-hydroxypyrazole core and different heterocyclic systems such as triazoles and oxadiazoles, linked to it [128]. In this way, they aimed to achieve a chemotherapeutic synergy, increasing the selectivity and decreasing the toxicity of the substances. It seems that the substituent at the 4-position of the 1,2,4-triazolin-3-thiones influences the antitumor activity, as well as in the case of the 1,3,4-oxadiazoles this is closely related to the nature of the substituent at 2-position. Thus, from the battery of synthesized compounds, 1,2,4-triazolin-3-thione **18** (4-cyclohexyl substituted) and 1,3,4-oxadiazoles **19** (2-one substituted) and **20** (2-phenyl substituted) (Fig. **15**), presented very good GI_50_ values for all the breast cancer lines tested: MCF-7, NCI/ADR-RES, HS 578T and MDA-MB-435, showing results below 0.01 μM in some cases. Among them, compound **20** showed an amazing profile of activity towards the rest of the cancer cell lines studied (26 lines) with GI_50_ values lying in the nanomolar concentration range (< 0.01 μM).

Interestingly, for further *in vitro* studies, compounds **18** and **19** were selected by the National Cancer Institute (NCI) Development Therapeutic Program and compound **20** was taken to the NCI Biological Evaluation Committee.

**Fig. (16):**
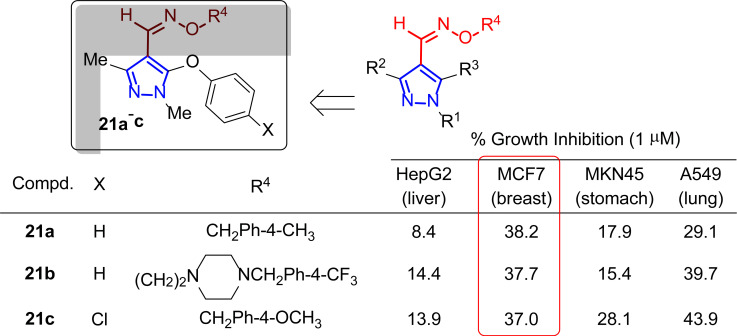
Pyrazole oxime ether derivatives **21** as potential antitumor agents [129].

In 2005, Lee’s group became interested in heterocyclic cores restricted to a limited number of conformations and hindered in rotation by the substituents [129]. This led them to synthesize pyrazole oxime ether derivatives and to evaluate their inhibitory potential against different tumor cell lines through sulforhodamine B (SBR) assay [130]. Cytotoxicity results were shown as a percentage of growth inhibition after adding the different compounds at 1 µM. Among the synthesized compounds differently substituted with alkyl or aromatic groups in the R^1^ and R^2^ positions, phenoxy in R^3^ and benzyl or 2-aminoethyl group in R^4^, only three of them **21a**-**c** (with R^1^, R^2^ = Me) had a moderate activity close to 40% inhibition at 1 μM against MCF-7 (Fig. **16**).

Analyzing the structure-activity relationship, a decrease in activity was observed when a larger group replaced the methyl group. Compounds with a benzyl group at the R^4^ position showed potent cytotoxic activity against a wide range of tumor cell lines, which led to further investigation of the modification at the R^3^ position of the pyrazole framework, although it was not tested in breast cancer lines.

**Fig. (17):**
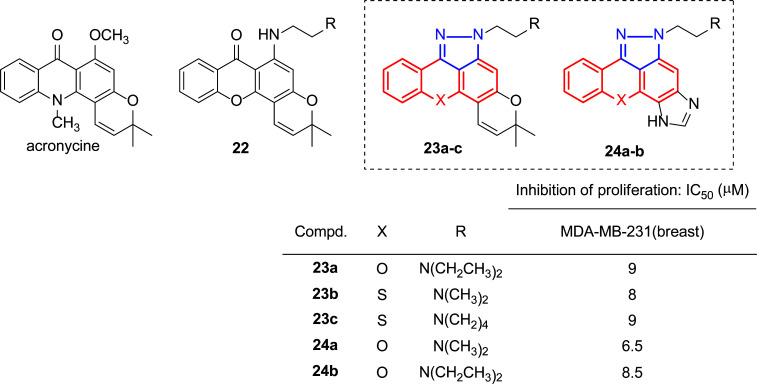
Structures of acronycine and prepared xanthenone derivatives **22**-**24** [140-142].

It is known that compounds based on a tricyclic planar structure consisting entirely or partially of anthraquinone, anthrapyrazole or acridine show interesting cytostatic and antitumor properties [131]. Furthermore, the presence of a five- or six-membered heterocyclic ring fused to the anthraquinone or acridine moiety can increase the activity and overcome the multidrug resistance of tumor cells [132, 133], there has been a growing interest in the development of these structures. Pouli’s group has worked on the synthesis and biological studies of pyrano(thio)xanthenone derivatives [134-136], possessing structural similarity to the pyranoacridone alkaloid acronycine (Fig. **17**), which showed promising antitumor properties in several murine solid tumor models [137] and was used as a lead for the synthesis of pharmacologically significantly improved analogues [138, 139]. A study of the structure-activity relationship in the pyranoxanthenone derivatives showed that substitution of the 6-methoxy group with a flexible (*N*,*N*-dialkylamino)ethylamino side chain substitution caused a strong enhancement of anti-proliferative activity towards the murine leukemia cell line L1210 (**22**, Fig. **17**) [140]. Furthermore, pyrazole-fused homologues of these molecules (pyran moiety: **23** [141] and imidazole ring: **24** [142], (Fig. **17**) were also synthesized and studied.

The results showed that incorporating a pyrazole ring in a xanthenone ring significantly increased antiproliferative activity. Fig. (**17**) presents the lowest IC_50_ (µM) values obtained against the MDA-MB-231 breast cancer cell line for these pyrazole-fused xanthenone amino derivatives.

**Fig. (18):**
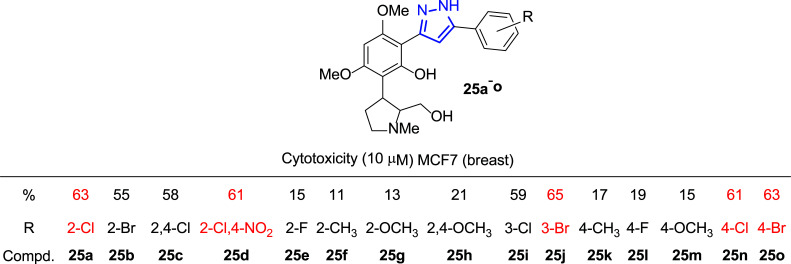
3,5-Diaryl pyrazole derivatives **25** synthesized for the biological assays [143].

At the same time, Bandgar and coworkers synthesized a library of 3,5-diaryl pyrazole derivatives **25** to test them against five tumor cell lines, including breast cancer (Fig. **18**) [143]. The screening method selected to carry out these assays was the fluorescence test with propidium iodide [144]. Dyes such as propidium iodide (PI), which bind to DNA, provide a quick and accurate tool to quantify total nuclear DNA. The intensity of the PI fluorescence signal is directly proportional to the amount of DNA in each cell. Propidium iodide cannot penetrate an intact membrane, so the cells must first be permeabilized; therefore, there must be a cellular damaged.

The best results regarding anticancer activity for the MCF-7 cell line were found with those compounds with the R substitution at positions 2 or 4 in the aromatic ring, preferably with Cl or Br substituents. Subsequent structure-activity studies revealed that only the halosubstituents (Cl and Br) were directly implicated in enhancing the anticancer activity. In contrast, fluorine, methyl and methoxy groups as substituents produced a marked decrease in activity (63% inhibition in the case of 2-Cl (**25a**) *versus* 15% for 2-F (**25e**), and 11% for 2-CH_3_ (**25f**) substitution, Fig. **18**).

However, it is not just the binding of the pyrazole structure to organic compounds that have been of interest in studying its biological properties. It is well known that ferrocene core has provided a versatile scaffold for the preparation of interesting structures useful in many areas such as catalysis, materials science, or bioinorganic chemistry [145-148]. In addition, numerous studies support that combining a ferrocenyl moiety with a heterocycle could increase their biological activity or give rise to new properties [149, 150]. Moreover, it has been demonstrated that ferrocenes could act in the activation of reactive oxygen species (ROS) formed in tumor cells, partially contributing to the antiproliferative effects of cancer cells [151-153]. On the other hand, ferrocenium salts were the first type of organometallic compounds reported to have antiproliferative properties, and nowadays, many ferrocene-based compounds are used as therapeutics [154, 155].

**Fig. (19):**
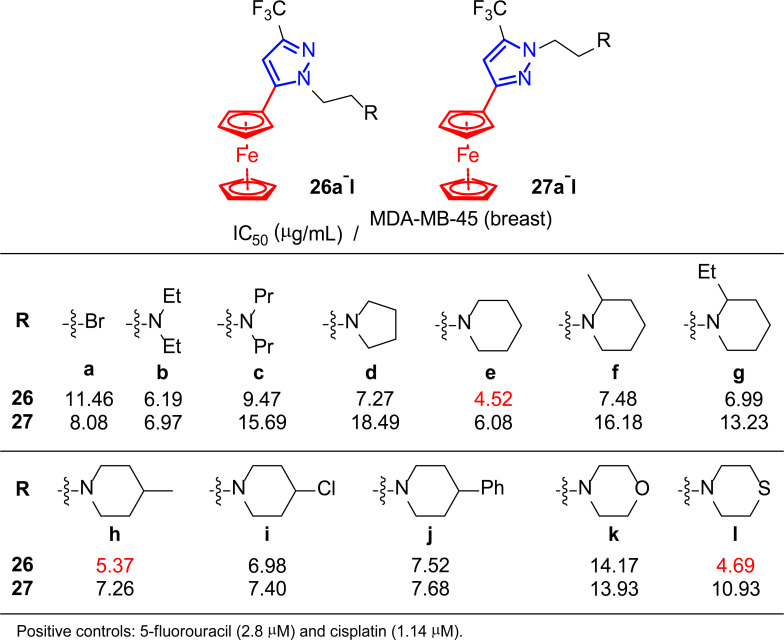
Ferrocene derivatives **26** and **27** used for the *in vitro* assays [158].

In this context, Ruan and co-workers, encouraged by their previous works [156, 157] and the properties of ferrocene derivatives, developed a variety of ferrocenes containing the pyrazole moiety **26** and **27** to study their activity as antitumor agents (Fig. **19**) [158].

*In vitro* assays were performed to study three tumor cell lines, including breast cancer MDA-MB-45, using 5-fluorouracil and cisplatin as positive controls (Fig. **19**). Although none of the synthesized compounds displayed IC_50_ values lower than these references, the results were below 9 μg/mL in most cases. In particular, compounds **26e**,**h**,**l** showed the strongest inhibitory activities against the MDA-MB-45 cell line, with IC_50_ values lower than 6 μg/mL. Therefore, these promising results, together with the low toxicity and the possibility of oral administration, positioned these compounds as good candidates for developing new anti-tumor drugs.

**Fig. (20):**
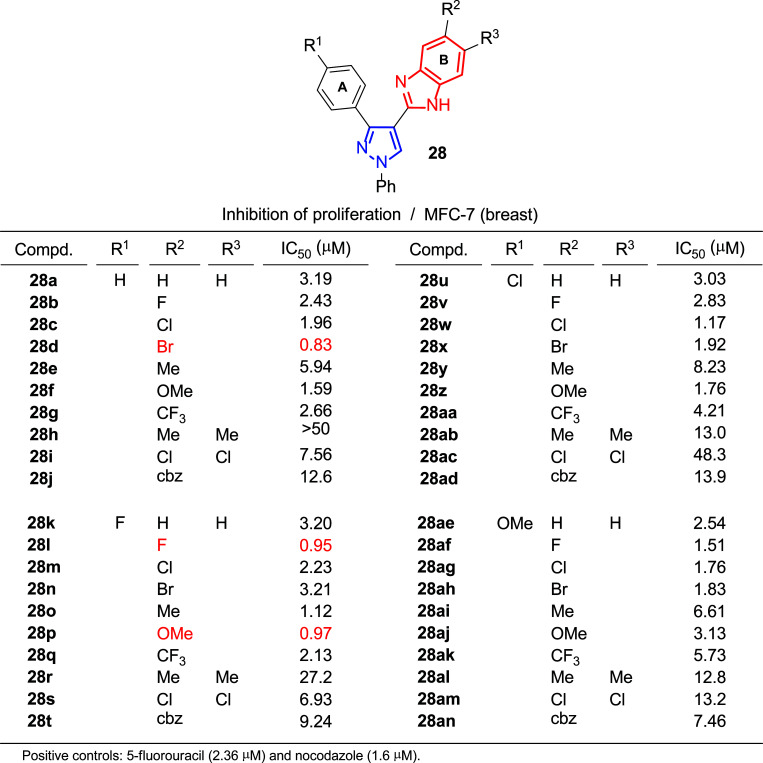
*In vitro* antiproliferative activity (IC_50_) of pyrazolo-benzimidazole derivatives **28** [166].

In search for new bioactive compounds, the pharmacophore hybridization strategy has been proven effective in modern medicinal chemistry since combining two molecules with different mechanisms of action often results in improved effects [159, 160]. In this context, and inspired by hybrid molecules containing benzimidazole (an interesting heterocycle for its biological properties and applications in medicine [161-163]) with enhanced anticancer activities [164, 165], Kamal, Shukla and coworkers undertook the synthesis of pyrazole derivatives **28** which incorporate that structural motif to produce promising new anticancer agents (Fig. **20**) [166].

The authors assessed the cytotoxicity of the prepared hybrids against HaCaT (normal keratinocyte cells) and three different cancer cell lines: A549 (lung), HeLa (cervix) and MCF-7 (breast, Fig. **20**). In general, growth inhibition was highly satisfactory, finding in many cases better results than those of controls used such as 5-fluorouracil (IC_50_ = 2.36 μM) and nocodazole (IC_50_ = 1.6 μM). In the case of the breast cancer line, values of IC_50_ below 1 μM were reached for several compounds (**28d**,**l**,**p**). In addition, the line corresponding to human epidermal keratinocyte provided numbers above 50 μM in most of the assays, which indicated selectivity of the pyrazoles **28** towards cancer cells in contrast to normal HaCaT cells.

Structure-activity relationships generally showed that mono-substitution with an F, Cl, Br and OMe group on the benzimidazole B ring exhibited more potent activities than CF_3_ groups and Me. In addition, di-substitution with Cl and Me groups demonstrated a lower inhibitory effect. On the other hand, it seems that substitution on the A ring did not influence significantly.

Moreover, the authors conducted further mechanistic studies with MCF-7 cells using the most potent compounds. For instance, it was found that the number of viable MCF-7 cells was significantly reduced after treatment with these compounds with concentrations corresponding to their IC_50_ for 48 h, compared to control cells.

Additional evaluation of the long-term cytotoxicity by measuring the ability of a single cell to grow into a colony revealed that these compounds inhibited MCF-7 cell colony formation, and all of them reduced clonogenic survival by about 50%.

As a potential metastatic measurement, the migration capacity of cancer cells is also a property of interest to be estimated. In this case, after treatment with pyrazolo-benzimidazoles, the amount of invasive breast cancer cells penetrating the respective wound areas was inhibited.

In addition, flow cytometry assays indicated that cell cycle arrest in the G0/G1 phase contributed to cytotoxicity. Studies conducted to elucidate the responsible mechanisms concluded that these compounds could decrease the levels of cyclins and cyclin-dependent kinases (CDKs), cell cycle regulators, leading to cell cycle arrest and, consequently, inhibiting the cell growth.

The apoptotic effect was studied through morphological changes found in MCF-7 cells treated with the IC_50_ concentration of the corresponding compounds for 24 h and after staining with Hoechst 33242. While control cells treated with DMSO showed uniformly dispersed chromatin, 25-34% of the cells treated with the different compounds showed apoptotic features, including chromatin condensation and nuclear degradation. Moreover, MCF-7 cells were treated with the IC_50_ concentration of the different compounds for 24 hours and chromosomal DNA was extracted to perform DNA fragmentation assays which may give extra information about the apoptosis-inducing effect of these products. The DNA-smeared ladder pattern observed was compatible with DNA breaks at multiple positions across the chromosomal DNA. Further contributions to the apoptotic effect of these compounds were identified, such as the 44-62% loss of mitochondrial membrane potential (DΨm) and the 2-4 times increased levels of reactive oxygen species (ROS) in the mitochondria, exhibited by compound-treated cells compared to control cells.

A failure to regulate apoptosis can lead to disease, including cancer. During this process, cysteine protease enzymes, called caspases, are activated, making caspase 3 a key regulator. Using those proteins as a target to induce apoptosis in cancer cells has emerged as an attractive strategy in cancer therapy [167]. Moreover, the introduction of a trifluoromethyl group, which increases the lipophilicity of the final products, has been of collective interest during the last decades for its effect on pharmacological properties [168]. Increased absorption across cell membranes improves the selectivity, efficacy and bioavailability of the compound in the body [169]. In addition, incorporating an azo group has also been of interest following the discovery of its ability to raise the biological activity of heterocyclic compounds [170].

**Fig. (21):**
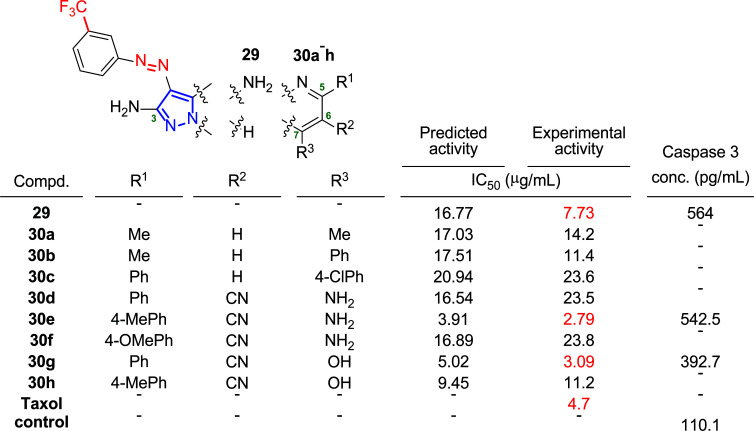
Molecular structures of **29** and **30a**-**h**, and results of the activity against MCF-7 cells: predicted activity, experimental activity and caspase 3 enzyme assay [171].

Given these comments, Fayed group has recently designed a new class of pyrazoles **29** and pyrazolopyrimidines **30** bearing trifluoromethyl and azo groups into their molecular structures to examine the effect of this combination on the anticancer activity of the system [171]. In particular, the authors studied three different cancer cell lines: HepG-2 (liver), HCT-116 (colon) and MCF-7 (breast). Fig. (**21**) shows the predicted and experimental activity expressed as IC_50_ values obtained for the latter cancer cell line. The efficacy of the most active compounds (**29**, **30e** and **30g**) in activating caspase 3 in the MCF-7 cell line was tested to elucidate the pathway leading to cell death through these new derivatives. Thus, treatment of the cells with **29** and **30e** resulted in an almost 5-fold increase in the level of caspase 3 compared to the control, whereas in the case of **30g**, this increase was only 3.6-fold, suggesting a caspase-dependent pathway to explain the activity of these compounds (Fig. **21**).

The further analyses of a structure-activity relationship based on modeling studies led to conclude that a CN group at the 6-position, a (substituted)phenyl group at the 5-position and a free NH_2_ group at the 2-position of pyrazolopyrimidine-containing compounds **30**, as well as the latter group at 3-position of the non-fused pyrazole scaffold **29**, could be a requirement for anticancer activity. Moreover, these studies confirmed the importance of fluorine building blocks in substituting the pyrazolopyrimidine scaffold.

**Fig. (22):**
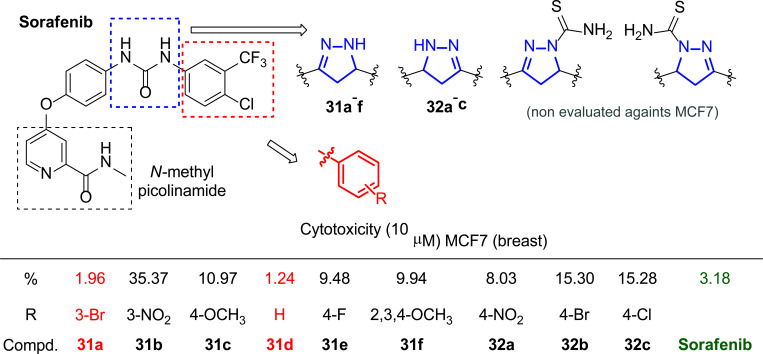
Sorafenib molecule-based substitution pattern developed [175].

An important aspect to consider in medicine is the concept of “drug repurposing” as an alternative to developing drugs from the ground up [172]. This approach provides the opportunity to find new drugs from pre-existing ones or molecules with proven good activities, which is a substantial time and cost saver. With this premise, Zheng, Zhu and coworkers decided to synthesize novel derivatives (**31** and **32**) of Sorafenib, bis-aryl urea with remarkable biological properties [173, 174], replacing this group with a pyrazole moiety to evaluate the influence of the change on the cytotoxicity against several cancer cell lines (A549, HepG2, MCF-7, and PC-3) and kinases (VEGFR-2/KDR, BRAF, CRAF, c-Met, EGFR and Flt-3) (Fig. **22**) [175].

The results of the biological tests showed that the substitution of the urea fraction by the pyrazole moiety did benefit the antitumor activity. Moreover, the different substitutions in the ring also involved a change in the cytotoxicity of the final compound. Regarding the results obtained for the breast cancer line MCF-7, among other observations, it was concluded that the presence of Br in the 3-position of the aromatic ring (**31a**) or the lack of substituent (**31d**) was the most effective pattern through which the best IC_50_ values were obtained (1.96 and 1.24 μM, respectively), even lower than Sorafenib (Fig. **22**). However, strongly electro-attracting groups, such as NO_2_ in the 3-position (**31b**), decreased activity (Fig. **22**).

To further explore the mode of binding of these compounds to vascular endothelial growth factor (VEGFR-2), molecular simulation studies were performed with compound **31a**. These analyses showed that the mode of binding of the synthesized compound **31a** produced a new H-bond with the ASN923 residues of the carbonyl group of the *N*-methylpicolinamide that was not present in the reference compound, playing an important role in the enhancement of the inhibitory potency.

## CONCLUSION

It has been proven that the pyrazole core and the infinite number of derivatives to which it can be linked represent a privileged structure of great pharmacological interest.

Their action as kinase inhibitors or potent antitumor agents against breast cancer lines (MCF-7 or MDA-MB-231, among others) is highly effective, reaching in some cases a level of efficacy compared to current treatments. The activity of these derivatives has not only been reflected in breast tumors but also in other cancer cell lines such as HeLa (cervix) or A549 (lung) and even as antimicrobial agents, reflecting the wide spectrum of biological activities that can be achieved.

Among the many publications collected, we highlight the preclinical *in vivo* assays with some of them, and the promising results obtained, encouraging us to continue working on these structures, providing a new pathway towards effective and less invasive therapies.

## Figures and Tables

**Table 1:** Molecular structures 7a-k and *in vitro* results for different breast cancer cell lines (Growth inhibitory concentration (GI_50_, µM)).

**Compounds 7a-k**		**MDA** **MB-231** **ATTC**	**MCF-7**	**T-47D**	**NCI** **ADR-RES**	**HS-578T**	**MDA** **MB-435**	**MiDA-N**	**BT 549**
**7a**	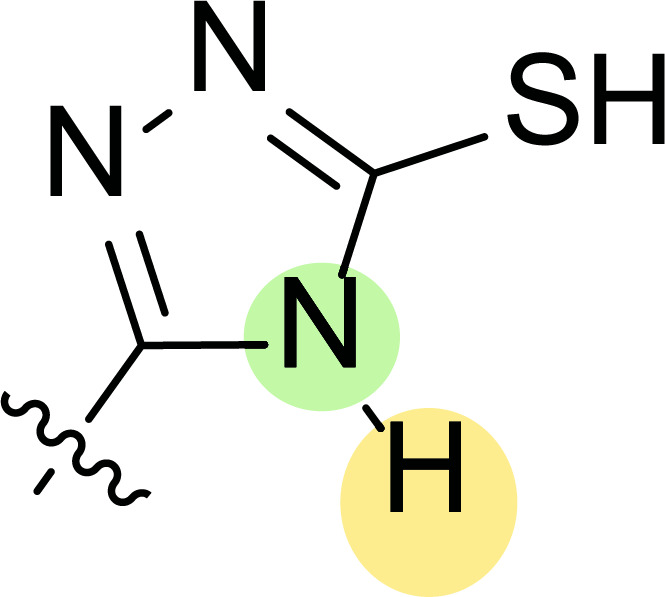	6.27	IA*	6.68	2.48	IA*	2.64	2.58	IA*
**7b**	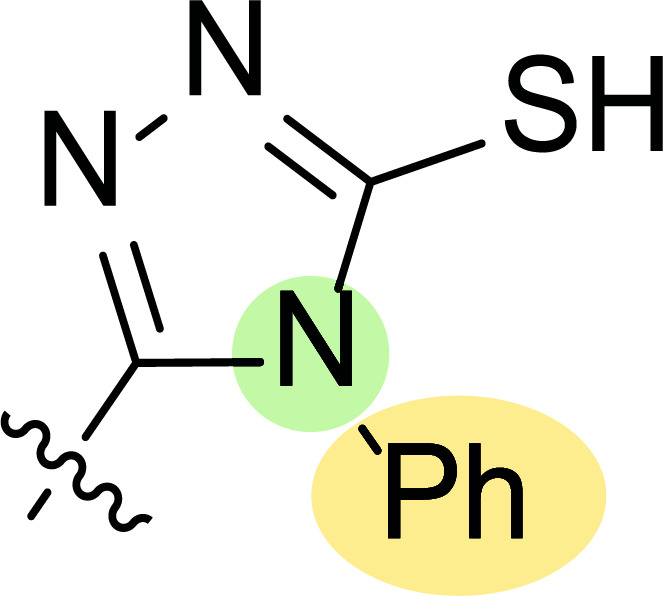	IA*	IA*	IA*	IA*	IA*	IA*	IA*	IA*
**7c**	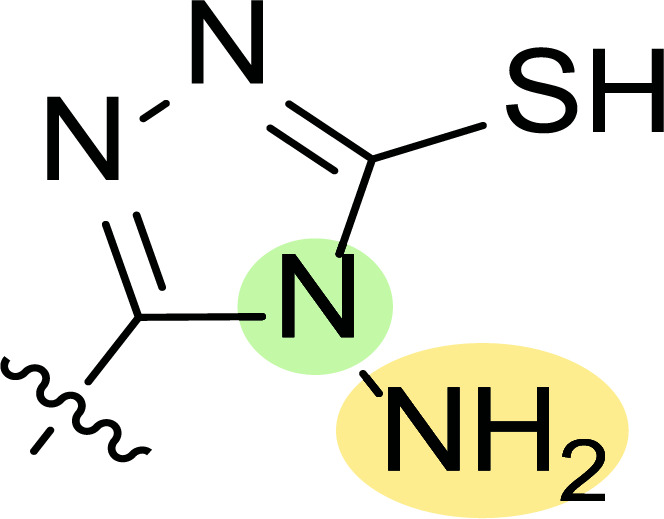	IA*	23.4	39.9	IA*	8.94	2.93	5.00	IA*
**7d**	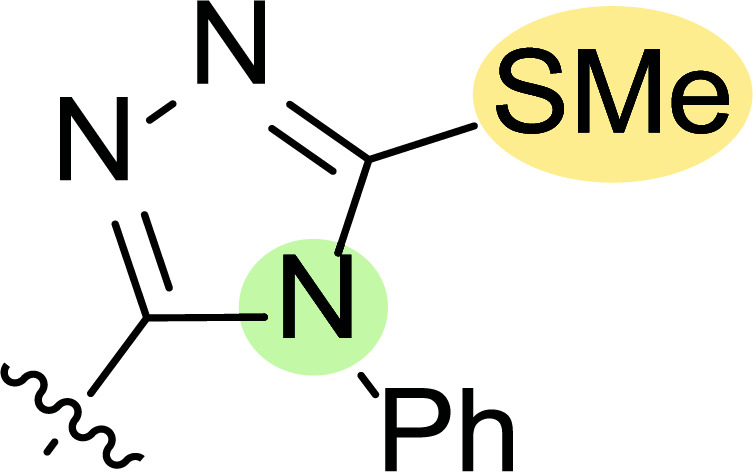	IA*	68.0	36.3	IA*	69.8	41.8	73.8	44.8
**7e**	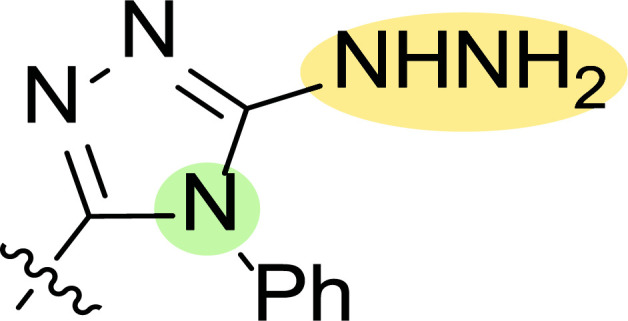	IA*	IA*	IA*	IA*	IA*	IA*	IA*	IA*
**7f**	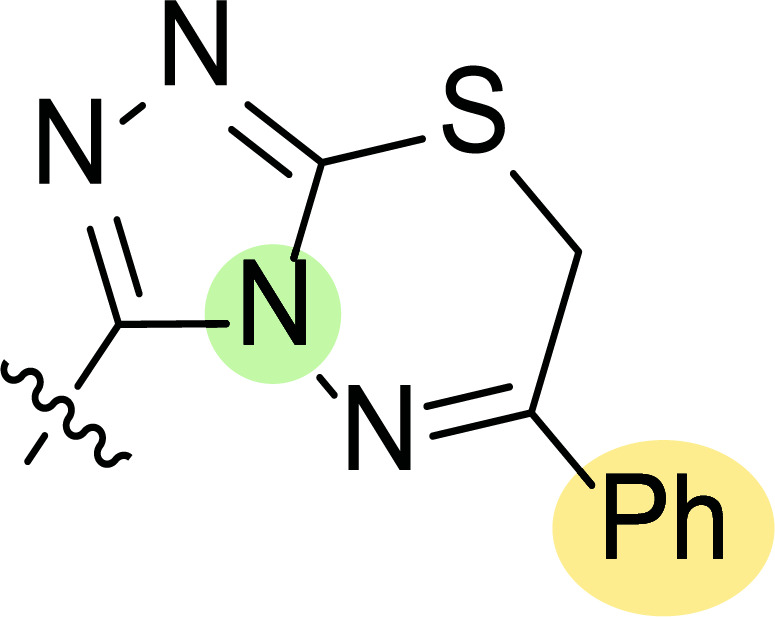	13.2	19.8	9.08	28.0	IA*	48.0	73.0	51.6
**7g**	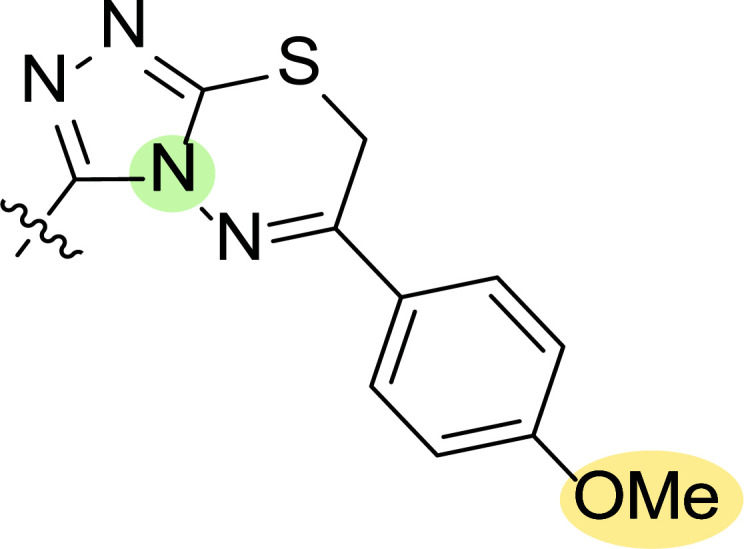	5.49	IA*	IA*	8.4	9.23	1.23	1.19	IA*
**7h**	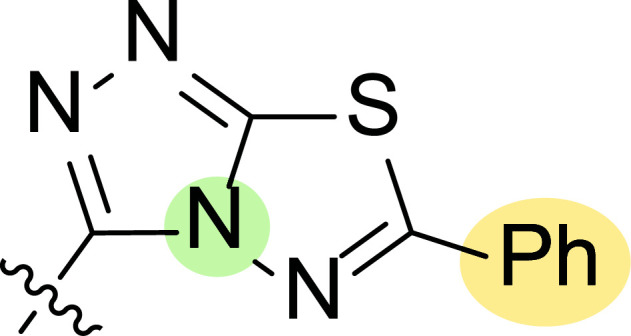	IA*	IA*	IA*	IA*	IA*	IA*	IA*	IA*
**7i**	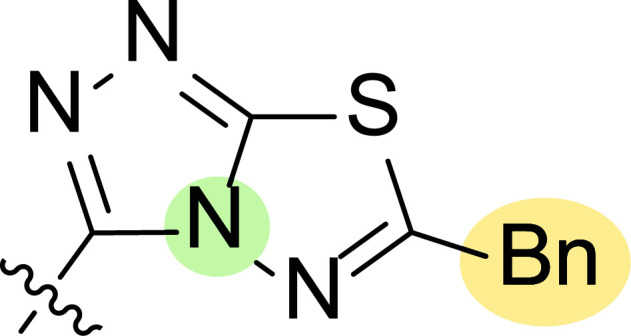	52.1	41.6	42.6	74.7	8.94	9.23	5.49	IA*
**7j**	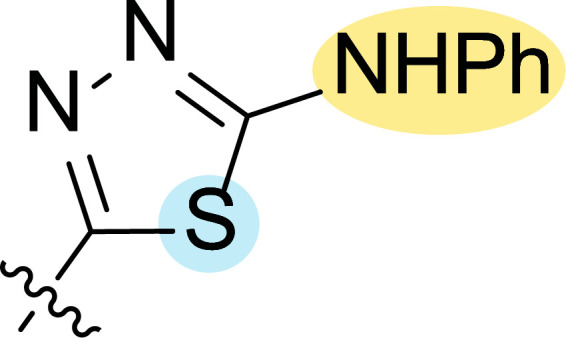	5.84	8.53	18.0	99.8	IA*	55.8	93.8	IA*
**7k**	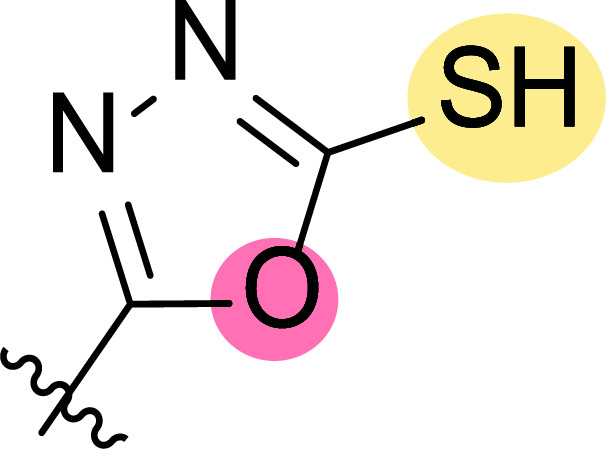	38.4	38.0	72.0	99.0	98.0	IA*	55.4	26.2

**Note:** *IA: Inactive; GI_50_ >100 µM.

**Table 2:** Molecular structures (8a-i) and *in vitro* results for different breast cancer cell lines (Growth inhibitory concentration (GI_50_, µM)).

**Compounds 8a-i**		**MDA** **MB-231** **ATTC**	**MCF-7**	**T-47D**	**NCI** **ADR-RES**	**HS-578T**	**MDA** **MB-435**	**MiDA-N**	**BT** **549**
**8a**	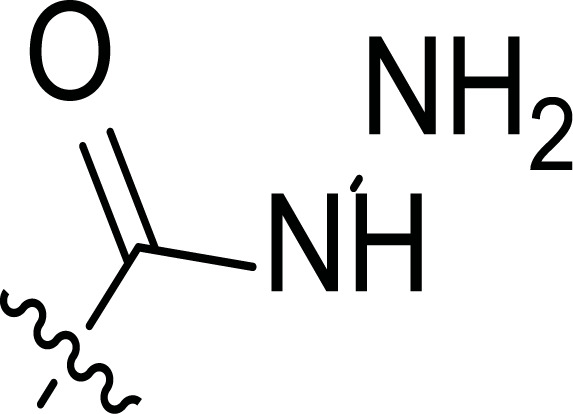	61.2	85.8	NT**	NT**	NT**	NT**	1.34	9.13
**8b**	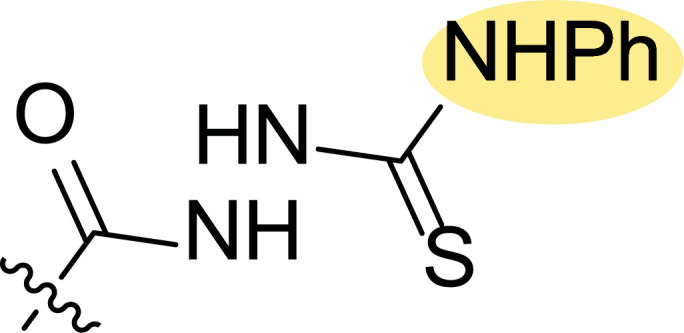	IA*	99.8	45.6	IA*	61.8	42.8	34.8	37.7
**8c**	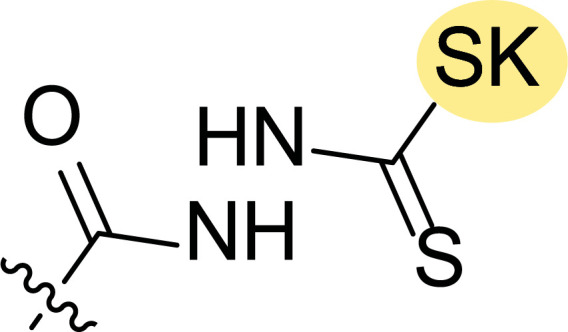	4.90	IA*	63.4	IA*	8.04	9.30	2.86	IA*
**8d**	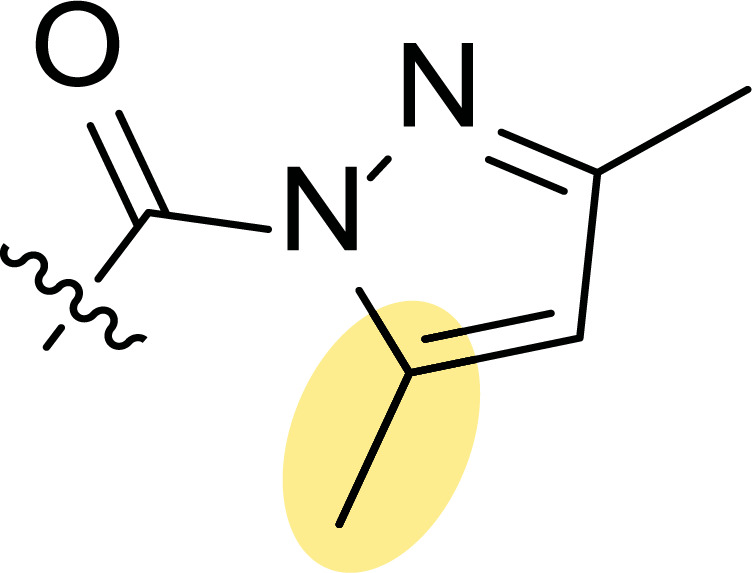	6.21	IA*	2.45	2.76	IA*	2.75	IA*	IA*
**8e**	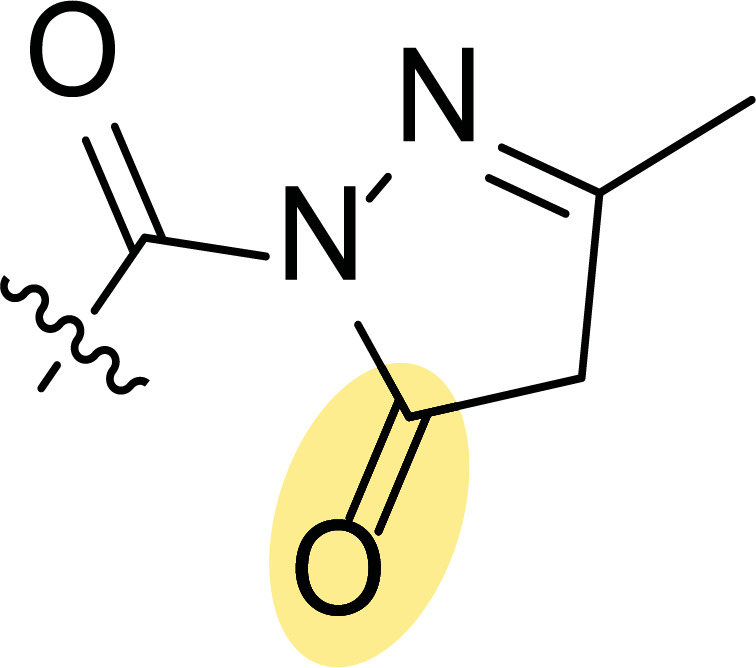	23.8	19.8	54.8	98.0	IA*	39.9	23.8	11.8
**8f**	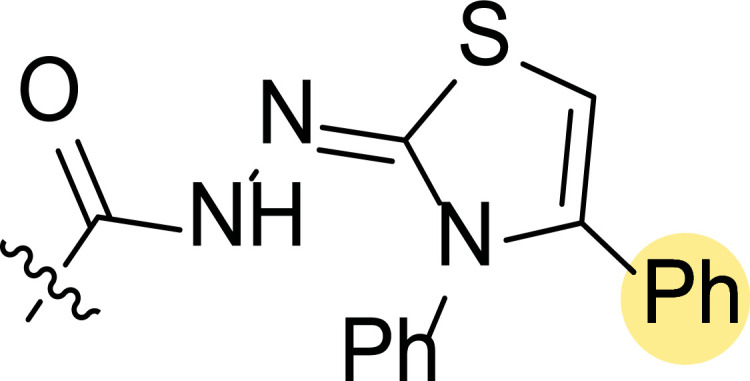	7.36	4.72	62.5	7.4	6.72	8.62	7.00	4.12
**8g**	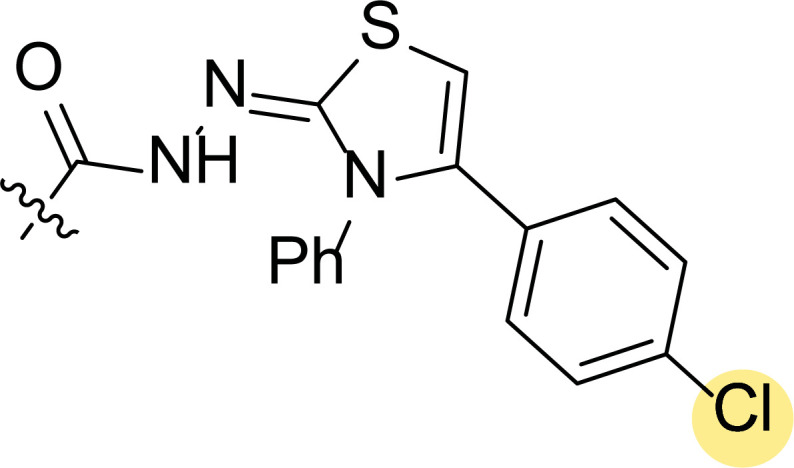	28.0	40.8	22.2	IA*	61.8	61.0	56.8	11.8
**8h**	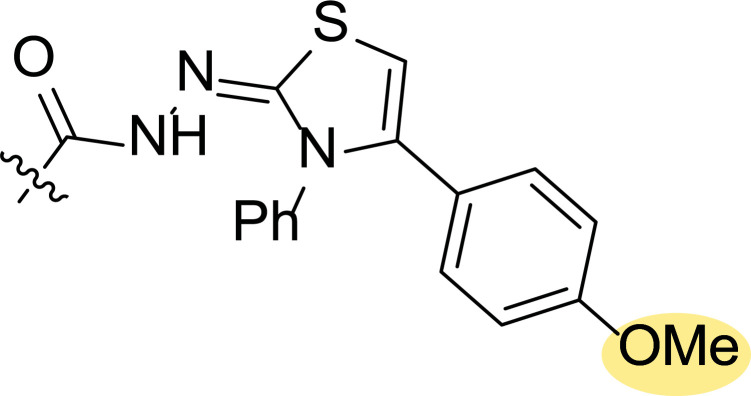	IA*	IA*	IA*	IA*	IA*	IA*	IA*	IA*
**8i**	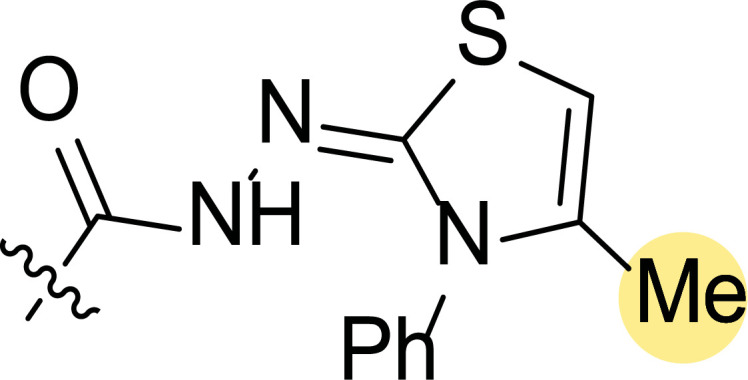	IA*	79.8	26.5	28.0	IA*	IA*	26.5	11.8

*IA: Inactive; GI_50_ >100 µM, **NT: not tested.

**Table 3:** Acute toxicity (LD_50_) of the synthesized compounds and letrozole.

**Compounds**	**LD_50_ (mg/kg)**
**Letrozole**	252.61 ± 0.11
**7a**	434.71 ± 0.13
**7b**	122.48 ± 0.13
**7k**	323.06 ± 0.11
**8a**	332.11 ± 0.13
**8b**	311.52 ± 0.12
**8c**	431.85 ± 0.13
**8d**	873.06 ± 0.19
**8e**	234.71 ± 0.13

## LIST OF ABBREVIATIONS

HDI: Human Development Index
SD: Sprague-Dawley
MDR: Multidrug Resistance
ECM: Extracellular Matrix
SRB: Sulforhodamine B
ROS: Reactive Oxygen Species
CDKs: Cyclin-Dependent Kinases
